# Increasing Quality and Quantity of Life in Individuals with Chronic Obstructive Pulmonary Disease: A Narrative Review with an Emphasis on Pulmonary Rehabilitation

**DOI:** 10.3390/life15050750

**Published:** 2025-05-07

**Authors:** Suzanne Lareau, Richard ZuWallack, Linda Nici

**Affiliations:** 1 College of Nursing, University of Colorado, Denver Anschutz Medical Campus, ED 2 North, 13120 East 19th Ave., Aurora, CO 80045, USA; 2 Pulmonary and Critical Care, Trinity Health of New England, St. Francis Hospital, 114 Woodland Street, Hartford, CT 06105, USA; 3 The Warren Alpert Medical School, Brown University, 222 Richmond St., Providence, RI 02903, USA

**Keywords:** COPD, survival, mortality, quality of life, HRQL

## Abstract

Goals of medical management of individuals with chronic obstructive pulmonary disease (COPD) should be to live better and live longer—in other words, improve health-related quality of life (HRQL) and survival. This narrative review summarizes the literature in these areas, with an emphasis on pulmonary rehabilitation (PR). Treatments that increase HRQL include pharmacologic agents, exercise training, physical activity promotion, lung volume reduction, PR, self-management training, and supplemental oxygen. Additionally, anything that reduces the frequency or impact of exacerbations substantially increases HRQL. With respect to survival in COPD, the list of beneficial interventions for this outcome is considerably more limited. Supplemental oxygen therapy for hypoxemic patients, smoking cessation interventions, influenza vaccination, and lung volume reduction procedures have the strongest evidence in survival benefit. PR, especially when provided following discharge for exacerbations, may improve survival. A nihilistic view of COPD treatment is unwarranted, as multiple interventions are available that improve HRQL, and likely increase survival for selected patients.

## 1. Introduction

Treating the individual with a serious, life-threatening chronic disease generally has two objectives: making that patient feel better and live longer; hence, the title of this narrative review: increasing both the quality and quantity of life. Meeting these objectives in chronic obstructive pulmonary disease (COPD) management generally requires both pharmacologic and non-pharmacologic interventions. Important physiologic markers of disease severity, such as airflow limitation, dynamic hyperinflation, and gas exchange abnormalities, are important physiologic endpoints to gauge therapeutic response. However, they are nonetheless surrogate markers for the two main objectives, increasing quality and quantity of life.

Most current therapies for COPD aim to improve health-related quality of life (HRQL), often by reducing the degree and impact of dyspnea, the overriding symptom in advanced disease. The effectiveness of pharmacologic and non-pharmacologic therapies on symptom burden and HRQL has been adequately demonstrated in the medical literature. Unfortunately, only a relatively small number of therapies for COPD have been shown to improve survival.

With respect to the interpretation of potential non-effects of various therapies on survival, an important caveat should be considered: *argumentum ad ignorantiam*, also known as an appeal to ignorance [1]. This is a logical fallacy that occurs when someone claims something is true or false because there is no evidence to the contrary. In other words, one should not necessarily assume the lack of a potential effect of a treatment for COPD if there is insufficient evidence to support it. In fact, it may be the case that the hypothesis has not been tested, there are insufficient data from underpowered studies, or substantial methodological issues are present.

## 2. Methods

This is an informal narrative review by authors with decades of academic and clinical experience in treating COPD and developing the science of pulmonary rehabilitation (PR). Scientific article selection was directed by the authors’ experience in the field, using PubMed and Google Scholar. The paper will briefly list therapies that have been demonstrated to enhance HRQL and increase survival in COPD. Following this, we will discuss the effects of comprehensive pulmonary rehabilitation (PR) on HRQL and survival in COPD [2]. Since the evidence for PR in improving HRQL in COPD is well-documented, we will focus in more detail on survival, including the quality of the available evidence and mechanisms that may explain a potential benefit in this area.

## 3. Chronic Obstructive Pulmonary Disease (COPD)

COPD, which is a leading cause of morbidity and mortality worldwide [3], has been recently defined as “…*a heterogeneous lung condition characterized by chronic respiratory symptoms (dyspnea, cough, expectoration) due to persistent abnormalities of the airways (bronchitis, bronchiolitis), and/or alveoli (emphysema), that often results in progressive airflow limitation*” [4]. Its diagnosis depends on objectively demonstrating airway obstruction (a forced expiratory volume in one second (FEV1) to forced vital capacity (FVC) ratio < 0.70) in an appropriate clinical setting.

As is the case with other chronic diseases, the morbidity and mortality burden of COPD reflects not only the primary disease process, but also its systemic effects and common comorbid conditions [5]. Prominently contributing to impaired quality of life in COPD are persistent and bothersome symptoms such as dyspnea, cough, and fatigue, respiratory exacerbations, high health care utilization, symptom-limited exercise intolerance, physical inactivity, social isolation, impairment in self-efficacy, and often burdensome anxiety and depression. Excess mortality in individuals with COPD results from both the underlying respiratory disease and comorbid conditions, especially cardiovascular disease and primary lung cancer.

While the most effective intervention to reduce the burden of COPD is its prevention through elimination of cigarette smoking and reduction in biomass smoke exposure, practicing clinicians must deal with the realities of treating established disease, its systemic effects, and common comorbid conditions [6]. Effective treatments should reduce overall disease burden and, if possible, slow the progression of the disease. Practically, this translates into both increasing health-related *quality of life* (i.e., feeling better) and *quantity of life* (i.e., living longer).

## 4. Health-Related Quality of Life (HRQL) in COPD

Quality of life, in general, is influenced not only by the disease process and its treatments, but also by the effects of other variables, including personality, economic status, environment, social relationships, and culture [7]. From a needs-based perspective, quality of life is good when most of the individual’s needs are fulfilled and poor when they are not [7]. This discussion will focus on a narrower concept—HRQL, which adds valuable, complementary information to other outcome measures, such as lung function. Since an individual’s perception of quality of life is unique to that individual, valid HRQL measures must get this information from that individual, as it cannot be inferred by health care providers or others. This has led to the concept of patient-reported outcome measures (PROMs), which typically are standardized, self-reported questionnaires assessing the patient’s perception of their health status. The use of PROMs is not limited to scientific investigation, but can be used clinically to assess patients’ perceptions and satisfaction [8]. Complementing PROMs are patient-reported experience measures (PREMs), although these are, to date, less utilized in COPD management [8].

Insight into factors that influence HRQL in COPD can be gleaned from three commonly used COPD-specific questionnaires that purportedly measure it. (1) The Chronic Respiratory Questionnaire (CRQ) [9,10] measures the domains of dyspnea, fatigue, emotion, and mastery; (2) the St. George’s Respiratory Questionnaire (SGRQ-C) [11] has three domains: symptoms, activity, and impacts; and (3) the unidimensional COPD Assessment Test (CAT) [12], assesses cough, phlegm, chest tightness, breathlessness, activity limitation, confidence, sleep, and energy. Summarizing the above, HRQL, as it pertains to COPD, reflects the impact of distressing symptoms, the psychological burden of the disease, the perceived ability to manage the disease (i.e., mastery or self-efficacy), and its effects on physical activity and participation. Of note, other components of disease burden, such as exacerbations, high health care utilization, and increased mortality risk, are not included in standard COPD-specific quality of life instruments, although these undoubtedly negatively influence scores on these instruments.

## 5. Improving HRQL in COPD

Table 1 lists categories of treatments that have been documented to improve HRQL in COPD. This list is by no means meant to be complete; rather, it is given to demonstrate the diversity of interventions that may improve HRQL in patients with this disease. In general, reducing symptom burden, increasing exercise capacity and physical activity, and decreasing the frequency or severity of exacerbations will lead to increased HRQL. Of note, the chronic care model, with its emphasis on organizing medical management across levels of care [13] and long-term supplemental oxygen therapy for hypoxemic patients with COPD, has had mixed results with respect to this outcome [14,15,16].

## 6. Survival in COPD

Worldwide, COPD is considered the fourth leading cause of death [17]. Unlike HRQL, which can be somewhat nebulous and subjective, assessment of mortality is more straightforward and can be measured using vital statistics data. The etiology of mortality can be reported as respiratory-specific or all-cause mortality (i.e., patients with COPD can die because of their disease or with their disease). This distinction can be difficult because comorbidities often play an important role in mortality risk [18]. Comorbidity, which is common in COPD, refers to an additional condition coexisting with the index disease [19]. The higher frequency of comorbid conditions in this disease is probably due to multiple factors, including shared etiologies. Comorbid conditions and systemic effects may add to COPD mortality risk through multiple mechanisms, including unhealthy lifestyle behaviors (e.g., smoking), direct effects of COPD on the heart, systemic inflammation, hypoxia, oxidative stress, impaired functional capacity, and skeletal muscle wasting and cachexia [20]. While some comorbid conditions are inconsequential with respect to mortality, others—such as lung cancer and ischemic heart disease—can markedly affect this outcome. Because of this, assignment to an all-cause mortality category is less fraught with problems in that one does not have to specifically identify which of the conditions (cardiovascular disease, cancer, etc.) in the COPD patient was responsible for their death.

## 7. Increasing Survival in COPD

Categories of treatments that may increase survival in COPD are given in Table 2. Again, this table is not meant to be exhaustive of all effective interventions in this outcome area, and for some categories, the supporting evidence is not substantial. Long-term supplemental oxygen therapy for hypoxemic COPD patients (PaO2 ≤ 55 mm Hg or ≤ 59 mm Hg with signs of right-sided heart strain or polycythemia) given for greater than 15 h per day is considered to have strong evidence for a survival benefit [21]. However, oxygen therapy for those COPD patients with less severe hypoxemia has not been shown to have a significant survival benefit [22]. Smoking cessation interventions, influenza vaccination, and lung volume reduction procedures for selected patients also seem to have robust evidence supporting a beneficial effect on survival.

The reason or reasons behind the survival benefit from interventions listed in Table 2 are not always clear. For instance, smoking cessation, especially when accomplished at a relatively young age, will slow down or even prevent the progression of airflow limitation characteristic of COPD [23,24], thereby reducing the risk of dying from this disease [23]. However, as demonstrated in a 14.5-year follow-up of the Lung Health Study, it also substantially reduced the risk of dying from other life-threatening conditions, including lung cancer, ischemic cardiac disease, and other respiratory diseases [25]. As another example, airflow limitation in COPD, manifested by a reduced forced expiratory volume in one second (FEV1), is a predictor of mortality in this disease. Since long-acting bronchodilators increase FEV1 in many patients, their beneficial effect in reducing mortality may be mediated, in part, via this mechanism. However, bronchodilators in COPD also reduce lung hyperinflation, which, in turn, increases intrapulmonary pressure and negative hemodynamic changes, potentially increasing mortality risk [26]. Adding to the difficulty in defining a specific mechanism in reducing mortality, long-acting bronchodilators also reduce the risk of respiratory exacerbations, which, in turn, impart a high mortality risk [27].

**Table 1:** Treatment categories that may have beneficial effects on health-related quality of life in COPD.

Category	References
Pharmacologic agents *	[28,29,30,31,32,33,34,35,36,37,38,39,40,41,42]
Exercise training	[43,44]
Physical activity promotion	[45]
Surgical and bronchoscopic lung volume reduction	[46,47]
Pulmonary rehabilitation Stable COPD Following exacerbation Tele-pulmonary rehabilitation	[48,49] [49,50,51] [52]
Self-management training	[53]
Chronic care model **	[54]
Supplemental oxygen therapy for hypoxemic patients **	[15,16,55]

* Includes long-acting beta agonists (LABA), long-acting muscarinic antagonists (LAMA), inhaled corticosteroids (ICS) (and their combinations), and phosphodiesterase inhibitors; ** Equivocal results.

## 8. Pulmonary Rehabilitation

Pulmonary rehabilitation is defined in a 2013 combined statement by the American Thoracic Society and European Respiratory Society as follows: “*Pulmonary rehabilitation is a comprehensive intervention based on a thorough patient assessment followed by patient-tailored therapies, which include, but are not limited to, exercise training, education, and behavior change, designed to improve the physical and psychological condition of people with chronic respiratory disease and to promote the long-term adherence of health-enhancing behaviors*” [56]. PR has little or no effect on lung function (other than indirectly decreasing dynamic hyperinflation); rather, its beneficial effects result from reducing the impact of systemic consequences and comorbid conditions associated with COPD. These include ambulatory muscle dysfunction, nutritional disturbances, psychological burden, cardiovascular deconditioning, and inadequate self-management skills [57]. Besides the effects of PR on systemic consequences and comorbid conditions, it can also promote integration of care, which is the seamless coordination of health care providers and systems across the continuum of the disease process [13].

As will be seen in the sections below, PR has a substantial effect on enhancing HRQL in COPD, and arguably a survival benefit, especially when given following hospitalization for exacerbations. Despite these benefits, it remains underutilized worldwide. For example, in a study of 223,832 Medicare patients discharged from the hospital following a COPD exacerbation—when they are at substantial risk for rehospitalization and mortality—only 1.9% and 2.7% initiated PR within 6 months and one year of their index hospitalization, respectively [58]. The reasons for this gross underutilization include non-referral by clinicians, unavailability of PR programs within a reasonable distance from the patient, reluctance on the part of the patient, comorbidity and disease severity, and low self-efficacy. Providing PR remotely (tele-pulmonary rehabilitation), which appears to have comparable effectiveness in some outcome areas [59], may alleviate some of this poor uptake.

The content of PR programs varies by health care system and individual program. A review of PR programs provided worldwide [60] lists interventions that may be given in PR: outdoor walking, treadmill walking, stationary cycling, resistance training, education and activities of daily living training, self-management training, nutritional support, inspiratory muscle training, neuromuscular electrical stimulation, smoking cessation, and psychological support. Of note, not all these interventions are given at a single site. One facet of PR is that the intervention is tailored to the needs and goals of the individual respiratory patient.

In most health care systems, pulmonary rehabilitation is provided in a center-based, outpatient program [61]. However, especially since the COVID-19 pandemic, there has been a trend toward tele-pulmonary rehabilitation (sometimes in hybrid form), and these novel interventions appear to confer similar benefits to center-based programs with respect to functional exercise capacity and HRQL outcomes [52,62]. Prolongation of survival, as an outcome, has not been tested with these newer models of pulmonary rehabilitation.

## 9. Increasing HRQL in COPD with PR

Randomized trials of outpatient, center-based PR demonstrate improvement in HRQL in COPD, with changes in this patient-reported outcome typically exceeding the minimal clinically important difference established for the measuring instrument [48]. Arguably, the magnitude of this effect is greater than that of any other treatment modality, including pharmacologic therapy [63]. The newer home-based and tele-pulmonary rehabilitation-based interventions lead to substantial improvements in HRQL compared to usual care, and appear to be comparable to traditional, outpatient PR interventions for this outcome [64,65,66].

## 10. Increasing Survival in COPD with PR

A limited number of studies suggest a potential beneficial effect of comprehensive PR on survival in COPD, as listed in Table 2. Most of these studies involved patients in the post-exacerbation period—a setting at high risk for subsequent mortality [67]. However, two retrospective audits conducted in the United Kingdom provide indirect evidence on a possible overall survival benefit from PR, not necessarily only involving those in the post-exacerbation setting. First, in a retrospective analysis of survival (among other outcomes) conducted at two hospitals within the University Hospitals of Leicester NHS Trust involving 1515 patients [68], 54.3% completed the program. Completers had significantly prolonged survival than non-completers (*p* < 0.001). The authors also showed that improvements of >50 m in the incremental shuttle walk test (a measure of exercise capacity) also resulted in improved survival. Second, in a National COPD PR Audit by the Royal College of Physicians (United Kingdom [69], 58% completed PR, resulting in a lower mortality rate than non-completers (8.3% vs. 12.3%, respectively, *p* < 0.001). Notably, in this second audit, the survival benefit remained even after adjustment for baseline case mix severity. Limitations to these retrospective audits include a potential baseline confounding of severity, and the fact that non-completion of PR may in itself be detrimental to one’s prognosis.

In the post-exacerbation setting, systematic reviews and meta-analyses have had mixed results with regard to PR and survival. One systematic review published in 2018 by Ryrsø et al., of four trials involving 319 patients [70], demonstrated a significantly reduced mortality: RR  =  0.58, 95% confidence interval: 0.35 to 0.98 (moderate quality of evidence). Conversely, a systematic review and meta-analysis published in 2024 [51] that included 9 studies involving 995 patients, found that PR post-hospital discharge failed to demonstrate a mortality reduction benefit (odds ratio 0.75, 95% CI 0.47 to 1.20, *p* = 0.24) (moderate quality evidence).

The above analyses underscore a major limitation with survival analyses: prospective, randomized studies are typically grossly underpowered to demonstrate benefit. In these instances, large databases can provide the power necessary to detect differences between cohorts. Two very large retrospective reviews of insurance databases [71,72], showed not only distressingly low referrals to PR but (importantly) positive and remarkably similar effects to Ryrsø et al. (2018) [70] on mortality risk (HR 0.63 and 0.67, respectively). The large retrospective cohort study by Lindenauer and colleagues [71], published in 2020, provides strong supportive evidence behind a survival benefit from PR following hospitalization for exacerbation. Of 197,376 U.S. Medicare beneficiaries hospitalized for COPD exacerbations, only 2721 (1.5%) initiated PR within 90 days of discharge. All-cause mortality was 19.4% within 1 year of discharge, again attesting to the poor prognosis in this setting. Using propensity modeling, mortality at 1-year in those initiating PR compared to those who did not participate in this intervention: hazard ratio 0.63 (95% CI, 0.57 to 0.69, *p* < 0.001). Furthermore, there appeared to be a dose-response relationship with additional sessions associated with a lower risk of death. As a non-randomized trial, this analysis might be affected by unmeasured confounding biases such as healthy survivor bias, however, severity and comorbid conditions were matched as best as possible. Despite this caveat, the striking findings, large numbers of subjects, and the real-world setting make these findings compelling.

**Table 2:** Treatment categories that may have survival benefit in COPD.

Category	
Long term oxygen therapy	[73,74]
Smoking cessation	[25]
Influenza vaccination	[75]
Lung transplantation	[76]
Surgical and bronchoscopic lung volume reduction	[46,77,78]
NIV in acute hypoxemic respiratory failure	[79]
Inhaled pharmacologic agents	[80,81,82,83]
Physical activity promotion	[84]
Pulmonary rehabilitation, supervised exercise training In stable COPD Following exacerbation	[68,69] [71]
Alpha-1 antitrypsin augmentation therapy	[85]

Notes on Table 2: Long-term supplemental oxygen therapy for hypoxemic patients, smoking cessation, influenza vaccination, and lung volume reduction have the strongest evidence. The ability of pharmaceutical agents (LAMA, LABA, ICS) in combinations to increase survival in COPD, despite large, randomized trials, is still controversial, especially since in most positive studies [80,81,82], this was not the primary outcome. However, a reasonable interpretation by Halpin and Fernandez in 2022 of the available data concluded that “…*pharmacotherapy has a beneficial effect on mortality in patients with more severe disease who are at risk of exacerbations, particularly those at risk of severe exacerbations. The findings are consistent across the studies, and there is a plausible mechanism underlying them*” [83]. Subsequently, a Cochrane review concluded that despite having a higher risk of pneumonia, all-cause mortality may be lower with triple therapy (i.e., LABA, LAMA, ICS) [86].

## 11. How PR May Improve HRQL and Survival in COPD

PR is an interdisciplinary, comprehensive intervention for individuals with chronic respiratory disease, involving patient assessment, exercise training, traditional education interventions, self-management training, and psychosocial support provided over several-weeks’ duration [2]. Ideally, PR is a major component of the integrated care of the patient with chronic disease, which includes: (1) providing the right care for the right patient at the right time, and (2) seamless coordination of care [13]. Optimal coordination of care is especially important following exacerbations of COPD [87], when HRQL and exercise capacity are markedly impaired, and risk for untoward outcomes, including rehospitalization and mortality, is substantially increased [88]. The above general description of PR and the importance of integrated care for the COPD patient provides a background to support its substantial benefit in HRQL and potential benefit in survival.

### 11.1. Rationale Behind the Positive Effects of PR on HRQL

Since PR has minimal or no direct effect on pulmonary physiology or lung function (apart from an indirect reduction in dynamic hyperinflation), changes in these parameters cannot explain its favorable effects on HRQL. Dyspnea and fatigue are the two most prominent symptoms in patients with COPD [89], and both can negatively impact HRQL in this disease. It is therefore no coincidence that two of the most used respiratory-focused questionnaires designed to measure HRQL in COPD, the CRQ and SGRQ [9,28], prominently address these symptoms and their impact.

Exercise training, a cornerstone of comprehensive PR [56], leads to dramatic improvements in exercise endurance [90] through several mechanisms, including physiologic adaptations in the muscles of ambulation [91], a physiologic training effect [92], and reductions in exercise-limiting dynamic hyperinflation [93,94]. Baseline performance on the six-minute walk test, a measure of functional exercise capacity, correlates significantly with baseline HRQL as measured by the SGRQ. However, after PR [95], the correlation between *improvements* in these two outcome categories (exercise performance and HRQL) is weak at best [96,97], indicating that HRQL is substantially influenced by other factors. These may include improvements in psychological function or symptoms, self-efficacy or disease mastery perception, other respiratory symptoms such as cough or wheeze, and fatigue. Fatigue—although common [98] and often debilitating in this disease—is arguably more nuanced than dyspnea, being influenced by multiple factors, including dyspnea, pain, anxiety, depression, and sleep [99]. PR leads to significant and clinically-meaningful improvements in fatigue [100].

Another potential mechanism, perhaps more circuitous, is the effect of PR in preventing the occurrence and reducing the impact of moderate to severe respiratory exacerbations and subsequent hospitalizations on HRQL [51,72,101]. HRQL is dramatically reduced in this setting [102]. How PR reduces severe exacerbations leading to this effect is not clear, but presumably, increases in exercise capacity and physical activity, better adherence to therapy and vaccination, improved self-management capability, and enhanced integrated care likely play a role.

### 11.2. Rationale Behind the Positive Effects of PR on Survival

The mechanism(s) of action for a potential survival benefit of PR in COPD are also not clear. Evidence supports the beneficial effects of PR on exercise capacity, dyspnea, HRQL, lean body and ambulatory muscle mass, and physical activity. Higher performance on each of the above outcomes, in turn, is associated with survival in COPD [103,104,105,106,107]. Is the putative beneficial effect of PR on survival mediated through improvements in some or all of these outcomes? Since correlation does not imply causation, the mechanistic basis remains uncertain.

It is noteworthy that the strongest evidence, by far, for a survival benefit with PR is when it is provided following hospital discharge for exacerbations. This underscores the severity of the exacerbation and the poor prognosis in this setting. The demonstration of reduced mortality following hospital discharge may reflect the lower statistical power required to show this beneficial relationship. Demonstrating a reduction in mortality in stable (not post-exacerbation) COPD would require a much greater number of subjects to achieve statistical significance, although the 2 UK audits described above support the contention that survival benefit may extend to this COPD population.

For stable COPD patients and those recovering from exacerbations, the PR intervention, typically intensive over several weeks, offers the opportunity to provide health-enhancing treatments that usual care does not. These include: (1) A comprehensive assessment which may identify previously undiagnosed, treatable, and potentially life-threatening conditions; (2) Exercise training, based on the capabilities of the individual patient, which should improve exercise capacity and activity levels that are associated with better survival; (3) Improved self-management education and skills, which should result in better medication and supplemental oxygen adherence and effective exacerbation action plans; (4) Enhanced engagement and support from the community [108], which may be associated with lower mortality [109]; (5) Increased engagement with the medical community, as a component of integrated care (although a survival benefit is inconclusive [110]); and (6) Reduction in frailty [111], which is associated with a higher mortality risk in COPD [112].

## 12. Conclusions

Optimal management of individuals with COPD should involve improving both the quality and quantity of life. Pharmacologic and non-pharmacologic interventions have demonstrated effects in these two areas. This narrative review focused on the effects of PR on both HRQL and survival. Strong evidence supports the beneficial effects of PR on HRQL, probably through reducing symptom burden, increasing exercise capacity, enhancing self-efficacy, and decreasing the impact of exacerbations. Current evidence supporting PR in reducing mortality is mixed, with two large, retrospective studies and one systematic review and meta-analysis showing positive effects, but one recent systematic review and meta-analysis not showing this benefit. If PR does improve survival, several mechanisms may be responsible for this effect. This comprehensive intervention with considerable time spent with the patient has the capacity to identify and address comorbid conditions, improve exercise performance and physical activity, increase engagement with the lay and medical community, enhance self-efficacy (including adherence with treatments and action plans for exacerbations), and decrease the impact of exacerbations.

## Data Availability

Not applicable.
